# Analysis of Antidepressant, Benzodiazepine Anxiolytic, and Hypnotic Use When Treating Depression, Anxiety, and Aggression in Pain Clinic Patients Treated for Neuropathic Pain

**DOI:** 10.3390/life12030433

**Published:** 2022-03-16

**Authors:** Marcin Kolacz, Dariusz Kosson, Ewa Puchalska-Kowalczyk, Malgorzata Mikaszewska-Sokolewicz, Barbara Lisowska, Malgorzata Malec-Milewska

**Affiliations:** 1 Ist Department of Anaesthesiology and Intensive Care, Medical University of Warsaw, 02-005 Warsaw, Poland; marcin.kolacz@wum.edu.pl; 2 Department of Anaesthesiology and Intensive Care Education, Medical University of Warsaw, 02-007 Warsaw, Poland; pu.ewka@gmail.com; 3 Clinic of Anaesthesiology and Intensive Care, The Children’s Memorial Health Institute, 04-730 Warsaw, Poland; mikaszewska@gmail.com; 4 Department Anesthesiology and Intensive Medical Care, National Geriatrics, Rheumatology and Rehabilitation Institute, 02-637 Warsaw, Poland; blisowska19@gmail.com; 5 Department of Anaesthesiology and Intensive Care, Medical Centre of Postgraduate Education, 00-416 Warsaw, Poland; lmilewski@post.pl

**Keywords:** neuropathic pain, depression, anxiety, aggression

## Abstract

Depression, anxiety, and aggression accompany neuropathic pain. Effective treatment of these comorbidities enhances the outcomes of pain management. Therefore, the study was designed to analyze the relationship between the intensity of depression, anxiety, and aggression and the pharmacotherapy applied in the daily practice of treating neuropathic pain. The aim of the study was to evaluate the frequency of using antidepressants (ADs), benzodiazepine anxiolytics (BDAs), and hypnotics, and the influence of administering these on the intensity of depression, anxiety, and aggression in patients diagnosed with neuropathic pain. A multi-center survey was conducted among 421 patients. An evaluation of the severity of depression, anxiety, and aggression was made using the Hospital Anxiety and Depression Scale—Modified Version (HADS-M). Among the patients treated due to neuropathic pain, ADs are used much more often than BDAs and hypnotics. Depression was well controlled, while anxiety was identified as a possible uncontrolled therapeutic problem in these patients, despite the correlation between the frequency of AD and hypnotics usage and the severity of anxiety. We also found that women show a higher level of intensity in both anxiety and depression, but this does not influence the frequency of their being administered ADs, BDAs, and hypnotics.

## 1. Introduction

The complex pathomechanism of chronic neuropathic pain and its wide spectrum of symptoms makes treatment difficult [1,2,3]. An important clinical aspect is the impact of chronic pain on cognitive and behavioral issues, changes in behavior, emotional or psychological disorders, and cognitive impairment [4,5]. Anxiety and aggression are frequent symptoms going hand-in-hand with neuropathic pain syndromes [6,7].

Neuropathic pain is also associated with lack of sleep, problems with falling asleep, and maintaining the continuity of sleep [8,9]. Depression is observed in 18% to 85% of patients suffering from chronic pain [10,11,12,13]. On the other hand, the prevalence of pain in depressed patients attending psychiatric clinics was 51.8% to 59.1% [10,13].

There are several national and international guidelines and recommendations for the treatment of neuropathic pain. Baseline management consisting of nonpharmacological treatment, such as psychology, physiotherapy, exercise, and massage, should be initiated early. Interventional methods, for example, neurostimulation or targeted drug delivery management, are also effective but should be considered at a later stage in treatment [14,15,16].

However, the primary form of neuropathic pain treatment is pharmacotherapy.

Medications from the first option rely on antidepressants (ADs), anticonvulsants (gabapentinoids), and topical analgesics (lidocaine, capsaicin). Combination therapy is recommended as a second option of treatment. ADs can be used simultaneously with gabapentinoid (gabapentin, pregabalin) and topical analgesics. At this stage, weak opioid and the serotonin and norepinephrine reuptake inhibitor—tramadol can be included. Third-line and subsequent therapies consist of other pharmacotherapy options and interventional techniques [17,18,19,20,21].

In the pharmacotherapy of anxiety disorders, besides ADs, some of the drugs used are also benzodiazepine anxiolytics (BDAs) [22]. BDAs and hypnotics prescribed in the therapy of sleep disorders [23,24].

ADs are a highly heterogeneous group. They comprise tricyclic antidepressants (TCAs), serotonin-norepinephrine reuptake inhibitors (SNRIs), selective serotonin reuptake inhibitors (SSRIs), and norepinephrine-dopamine reuptake inhibitors (NDRIs) [25,26].

In neuropathic pain pharmacotherapy, TCAs (amitriptyline and nortriptyline) and SNRIs (duloxetine and venlafaxine) are used in first- and second-line treatment. SSRIs can also be considered, but this group is only recommended in third-line therapy [17]. They are also used for treating mental disorders, especially depression, anxiety disorders, phobias, and neurasthenia.

Benzodiazepines are a group of drugs with anxiolytic, sedative, hypnotic, anticonvulsant, muscle relaxant, and amnestic effects, which work by means of influencing GABAergic transmission via the benzodiazepine receptor. Particular drugs differ in their affinity to the receptor, their strength, and the profile of how their work.

The term hypnotics refers to those pharmaceuticals analyzed in the present study whose function is to modulate sleep: prolong it, make it easier to fall asleep, and reduce the frequency of waking up. The main groups of such medications are comprised of imidazopyridines and cyclopyrrolones. They function via binding to the benzodiazepine GABA-A receptor complex. This mechanism is probably connected to a lower risk for addiction than in the case of benzodiazepines [27,28].

Increased levels of depression, anxiety, and aggression accompanying neuropathic pain have an impact on the patients’ quality of life. This negative correlation decreases during pain therapy; however, effective treatment of these comorbidities enhances the outcome of pain management [29,30].

Therefore, the present multi-center study was designed to analyze the practical relationship between the intensity of the above comorbidities and the pharmacotherapy used in a group of patients with neuropathic pain treated on an outpatient basis in pain clinics.

The aim of the present study was to analyze the use of ADs, BDAs, and hypnotics, and to evaluate the influence of their use on the severity of anxiety, depression, and aggression in patients diagnosed with neuropathic pain who were treated in a pain clinic.

The primary endpoint of the study was to analyze the frequency of using ADs, BDAs, and hypnotics among pain clinic patients treated for neuropathic pain. The secondary endpoints were: assessment of the groups of drugs analyzed regarding the aspect of gender; assessment of the intensity of depression, anxiety, and aggression in the study group; and determining the relationship between the groups of drugs analyzed and the intensity of anxiety, depression, and aggression.

## 2. Material and Methods

Ethical approval for this study was provided by the Bioethical Committee at the Medical University of Warsaw, Poland, with the reference number AKBE/24/15.

The trial was conducted between January 2014 and April 2018 among the patients of two pain clinics: the 1st Department of Anesthesiology and Intensive Care, Medical University of Warsaw, Poland, and the Department of Anesthesiology and Intensive Care, Centre of Postgraduate Medical Education, Warsaw, Poland.

All patients 18 years old and older who were diagnosed with neuropathic pain on the basis of the guidelines of the Polish Society for the Study of Pain and had neuropathic pain questionnaire (DN4) scores equal to or greater than four, were not subject to interventional methods of pain treatment, and did not require simultaneous treatment of chronic pain of a different type, were considered for the study [20]. The patients were informed that their participation was voluntary and anonymous, and the results would be used solely for scientific purposes. The exclusion criterion was a lack of informed consent on the part of the patient. Simultaneous use of other forms of neuropathic pain pharmacotherapy did not exclude from the study.

The research intervention, consisting of filling out the authors’ questionnaire, was carried out once in the case of each patient who had been enrolled, irrespective of the period of time they had been treated at the clinic. The survey included data on the patient’s gender, the ADs, the hypnotics and BDAs taken, and the HADS-M scale. It assessed the state for the week preceding the survey.

The intensities of anxiety, depression, and aggression were assessed on a modified Polish version of the HADS-M (Hospital Anxiety and Depression Scale—Modified Version). It is composed of three elements. The subscale of anxiety and depression comprises seven elements, while the subscale of aggression is composed of two statements. The answers given are awarded from 0 to 3 points. The maximum number of points for the anxiety and depression scale is 21, while that for the aggression subscale is 6 points. In accordance with the suggestion of the authors of the HADS-M scale, the analysis of symptom intensity used the following thresholds: for anxiety and depression 0–7 points, no disorders 8–10 points, while borderline conditions >10 points indicated that disorders were found. For aggression, the point classes were: 0–2 points, 3 points, and 4–6 points, respectively [31].

All the data collected in the course of the study were gathered into the Microsoft Excel program of MS Office 2010 for Windows 10. Statistical analyses were performed using STATISTICA version 13 (StatSoft, Krakow, Poland). Qualitative data were described by means of quantity and percentage, while for qualitative data, median, and standard deviation (SD) were used.

## 3. Results

From 1 January 2014 to 31 April 2018, a total of 421 patients were identified for the study. Informed consent for participating in the research was obtained from all the patients included. All of the 268 women and 153 men involved completed the study.

The following drugs were taken by the groups analyzed: antidepressants (TCAs (amitriptyline, doxepin), tetracyclic antidepressants (mianserin), SSRIs (citalopram, escitalopram, fluvoxamine, and sertraline), SNRIs (venlafaxine, duloxetine, and trazodone)), benzodiazepines (diazepam, alprazolam, estazolam, and clonazepam), and hypnotics (zolpidem and zopiclone). The other drugs used in neuropathic pain management taken were: anticonvulsants (gabapentin, pregabalin, and carbamazepine), topical drugs (lidocaine, capsaicin patch), tramadol, and opioid analgesics (oxycodone, buprenorphine, methadone, fentanyl, and morphine).

**Figure 1:**
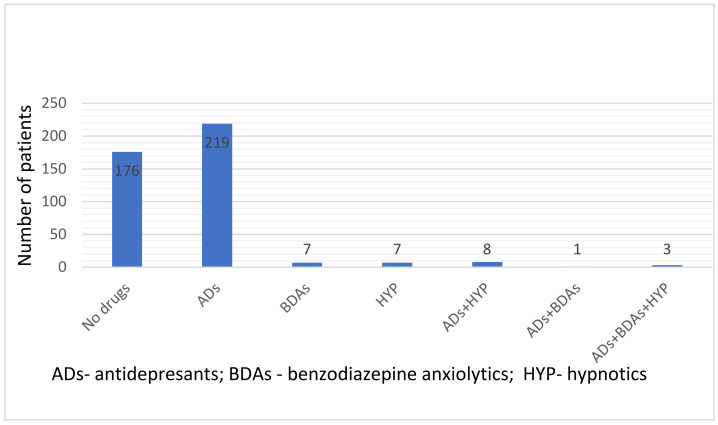
Groups of drugs under analysis taken by the patients enrolled in the study *n* = 421.

In the group that was analyzed, 231 (55%) of the patients took ADs. Out of these, this was their only drug for 219, while a hypnotic was also used by eight patients. One patient also took a BDA, and three patients additionally took two drugs, a hypnotic and a BDA. In monotherapy, both a hypnotic and a BDA were used in seven patients. Among the patients included in the study, 176 (42%) did not use any of the drugs from the groups under analysis (Figure 1).

**Figure 2:**
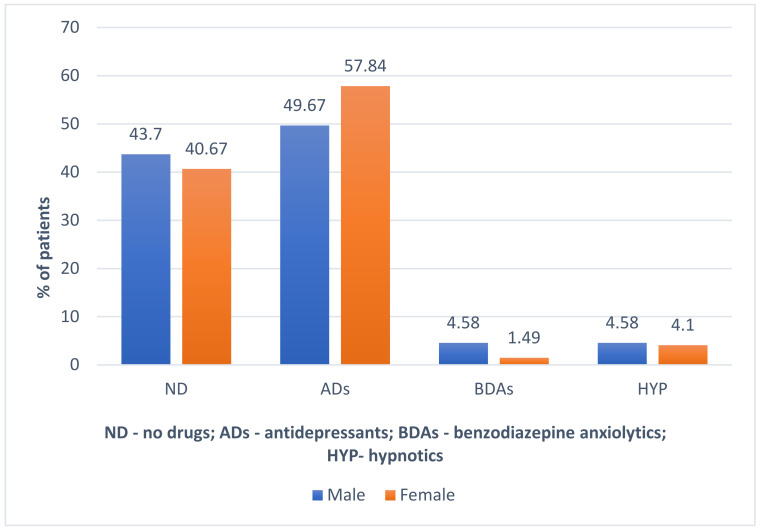
Dependence between the gender of the patients studied and the groups of drugs under analysis that were taken.

No statistically significant differences were found in the way the groups of drugs under analysis were taken depending on the gender of the patients (Table 1, Figure 2).

The intensities of anxiety, depression, and aggression were assessed in the analyzed group of patients. Most of them presented with borderline values for anxiety. The median for depression was close to the borderline values on the HADS-M scale. The scale did not show the presence of aggression in the analyzed group. The median scores for all the patients (*n* = 421) were: 8 (0–21) IQR 5–11 for anxiety; 7 (0–21) IQR 4–9 for depression; and 3 (0–6) IQR 2–4 for aggression.

It was shown that women show higher levels of intensity regarding anxiety and depression. There was no relationship between gender and aggression (Table 2).

After analyzing the relationship between the drugs administered and the intensity of anxiety, depression, and aggression on the HADS-M scale, it was demonstrated that antidepressants and hypnotics were more often used in patients diagnosed with anxiety (Table 3 and Table 4). This relationship was not observed for BDAs. No relationship between the intensity of depression and the frequency of using antidepressants, hypnotics, and BDAs was observed. As no aggressive disorders were found in the studied group, we did not investigate the correlation between this kind of behavior and the drugs analyzed.

## 4. Discussion

The aim of the analysis was to assess the frequency of using ADs, BDAs, and hypnotics and their influence on the intensity of depression, anxiety, and aggression in patients diagnosed with neuropathic pain. The study was multi-center, and it was conducted among patients treated in pain clinics.

In the literature available, Dutch analyses from 1996 to 2003 show that in the treatment of neuropathic pain, BDAs were used in 11.9%, hypnotics in 9.1%, and TCAs in 4.7% of the cases [32]. The present study shows a much lower use of both BDAs and hypnotics (2.6% and 4.3% of the cases, respectively), which might be related to progress in neuropathic pain management, as well as to the potentially dangerous side effects of administering them in conjunction with other drugs used for chronic pain therapy, particularly opioids, which is stressed in the recent literature [33,34].

In addition, the small percentage of combination therapy using the analyzed drugs (2.6%; *n* = 12 patients) (Figure 1) indicates that therapy with hypnotics and BDAs was limited in the present study.

A later, large-scale European cross-section study focusing on neuropathic pain found that 29% of the patients were prescribed antidepressants [35]. Simultaneously, in his 1992 study on the epidemiology and treatment of depression, Smith indicated that depressive disorders occur in about 50% of the patients suffering from chronic pain [36]. More recent studies confirm these results. Depression is also commonly seen in patients with neuropathic pain [37,38].

The present study shows that ADs were taken by 55% of the patients both in monotherapy and in therapy combining BDAs with hypnotics, which, given Smith’s findings, suggests that they were used primarily due to depression as a co-morbidity of neuropathic pain. This observation stays in line with the Cherif study. Using DN4 and HADS scales, the authors found that in the neuropathic pain population of patients, 65.57% present with symptoms of depression [13].

At the same time, some of the groups of antidepressants are among the potential choices in the pharmacotherapy of pain, which might suggest their other uses in managing neuropathic pain than the treatment of depression [20,39]. In the study being discussed here, 42% of the patients did not take any of the drugs from the groups under analysis (including ADs), despite the diagnosis of neuropathic pain. They could, however, have been given an alternative effective treatment option for neuropathic pain, in line with recommendations.

ADs are effective in managing depression, irrespective of whether it is a co-morbidity of the chronic pain syndrome [18,40,41,42].

In the present study, the intensities of depression, anxiety, and aggression on the HADS-M scale were discussed. Most patients were characterized by values showing borderline states of anxiety. The median for depression was below the borderline states on the HADS-M scale. Therefore, the frequent use of ADs demonstrated in the present study could have had an impact only on the effectiveness of the therapy for depression and not on the intensity of anxiety, despite the statistically significant correlation between the frequency of taking ADs and the severity of anxiety.

However, we show that the connection of disorders due to depression and anxiety with chronic pain is equally strong, while the dosage regimens of SSRIs, SNRIs, and TCAs in managing anxiety and pain are not much different. BDAs are also used in the treatment of anxiety. However, in this study, no relationship was found between the intensity of anxiety and the frequency of taking BDAs [18,20,42].

On the other hand, in the present study, it was proven that hypnotics were taken more often by those patients who were diagnosed with anxiety disorders on the HADS-M scale. According to the existing literature, hypnotics treatment in anxiety is not recommended [17,43].

However, this might be due to the FDA-mandated restriction of BDA use in opioid-treated patients. Such treatment is an option recommended in neuropathic pain management [20,44,45].

The assessment of the studied group of patients did not show values of significantly intense aggression among the patients treated due to neuropathic pain. Therefore, we did not investigate the correlation between aggression and the ADs, BDAs, and hypnotics taken.

The above results do not correlate with the available literature, because in neuropathic pain, aggression is described in both humans and in animals [6,46]. The frequency of its occurrence in cases of chronic pain is as high as 70% [8]. Such differences can indicate limitations of the HDS-M scale in identifying aggression in patients treated for neuropathic pain.

In addition, the effectiveness of pharmacotherapy in aggressive patients has not been supported by strong scientific evidence [47,48].

In the group of patients from the study under discussion, it was shown that women experience a higher intensity of anxiety and depression. No dependence due to gender was noted for aggression. These observations are consistent with the reports of other authors [5,49]. An equal rate of reported depression (male 12.3%, female 11.5%) was observed in the Swedish population. However, in the same study, the authors note a higher rate of Ads prescribed for women (9.8% vs. 5.3%) [50].

These results are corelated with other observations. According to Debra J Brody’s report conducted between 2015 and 2018, in the United States 13.2% of adults (18 years old and older) used antidepressant medications in the past 30 days. AD use was higher among women (17.7%) than men (8.4%) [51].

At the same time, in the group of patients from the study under discussion, no statistically significant difference was found when comparing genders for how the Ads were taken. Gender differences in the rate and intensity of depression and Ads taken may be caused by the alleviation of neuropathic pain in the diagnosed group of patients considered for the study under discussion. This group of patients may not reflect the trend in the overall population of patients treated for depression disorders.

In the AD group of drugs, the efficacy in neuropathic pain management is documented for TCAs and SNRIs. At the same time SSRIs are considered less effective in neuropathic pain treatment [52,53].

In the literature, it has been observed that gender has an impact on the type of antidepressant prescribed for treating depression disorders. Women use SSRIs more often and respond to them better than men. On the other hand, TCAs have been prescribed more frequently for men and are considered more effective in the treatment of depression disorders in this group of patients [54,55].

The above observations may favor men in the group of patients treated for neuropathic pain in terms of efficacy in the management of depression disorders, since TCAs are used more frequently in pain treatment (and recommended as the first- or second-line pharmacotherapy), and then SSRIs (the third line) are used [14]. This can also explain the higher intensity of depression experienced by women in the study under discussion.

In the group of patients from the study under discussion, it was shown that women, apart from depression, experience a higher intensity of anxiety. This observation stays in line with the available literature. The National Comorbidity Survey conducted in the United States from 1990 to 1992 found that lifetime prevalence rates for any anxiety disorder were 30.5% for women and 19.2% for men [56].

### Limitations

The HADS-M scale used in the study is a screening tool. It was originally designed for hospitalized patients, but is now also used to assess the emotional state of outpatients. The HADS-M scale was chosen because of its simplicity, which makes it possible to apply this instrument for a larger group of patients, while remembering its origin as a screening tool. Moreover, the small number of patients taking BDAs and hypnotics (in comparison with ADs) also has an impact on the results of the study. Therefore, our conclusions should be validated by subsequent research. The study did not assess particular medications, but only groups of drugs, which might have had an influence on the results of the analysis, considering the differences in their application.

## 5. Conclusions

In traditional conceptualization of pain treatment, the reduction in pain intensity is the main goal of management [57]. The molecular neurobiology of chronic pain induces depression and anxiety [58]. Neuropathic pain and its duration positively corelates with the intensity of mood disorders and has a negative association with quality of life, despite the level of pain intensity [13,59,60].

We found that among the patients treated due to neuropathic pain, ADs are used much more often than BDAs and hypnotics. Depression was well controlled, while anxiety was identified as a possible uncontrolled therapeutic problem in these patients, despite the correlation between the frequency of AD and hypnotics usage and the severity of anxiety. We also found that women show a higher level of intensity in both anxiety and depression, but this does not influence the frequency of their being administered ADs, BDAs, and hypnotics.

This finding suggests the need for evaluating the emotional state of patients treated for neuropathic pain. Selected ADs could possibly be considered as a first choice of pharmacotherapy in a depressed and anxious patient. The limited influence of the pharmacotherapy applied on the anxiety level in this group of patients suggests the need to put more emphasis on psychological treatment options. These options should also be considered more widely in neuropathic pain treatment in women.

## Figures and Tables

**Table 1:** Dependence between the gender of the patients studied and the groups of drugs under analysis that were taken.

	ND	ADs	BDAs	HYP	Total (n)
Male	67 (43.7%)	76 (49.67%)	7 (4.58%)	7 (4.58%)	153 (100%)
Female	109 (40.67%)	155 (57.84%)	4 (1.49%)	11 (4.10%)	268 (100%)
*p*	*p* = 0.79	*p* = 0.11	*p* = 0.06	*p* = 0.82	421

ND—no drugs; ADs—Antidepressants; BDAs—Benzodiazepine anxiolytic; HYP—Hypnotics.

**Table 2:** Dependence between the gender of the patients studied and the intensity of anxiety, depression, and aggression on the HADS-M scale.

	Median M (*n* = 153)	Median F (*n* = 268)	U	Z	*p*
The intensity of anxiety on HADS-M *	7	9	15,943.00	−3.80	0.00
The intensity of depression on HADS-M *	6	7	18,078.50	−2.02	0.04
The intensity of aggression on HADS-M *	3	3	20,104.00	0.33	0.74

* The intensity of anxiety disorders on HADS-M: 0–7 points, no disorders; 8–10 points, borderline states; >10 points, disorders found [31].

**Table 3:** Antidepressants (ADs) used and the intensity of anxiety.

The Intensity of Anxiety (HADS—M) *	Without ADs N (%)	ADs N (%)	Total	*p*
0–7 points	82 (43.16%)	97 (41.99%)	179	*p* = 0.04
8–10 points	58 (30.53%)	50 (21.65%)	108	
>10 points	50 (26.32%)	84 (36.36%)	134	
Total	190	231	421	

* The intensity of anxiety disorders on the HADS-M scale: 0–7 points, no disorders; 8–10 points, borderline states; >10 points, disorders found [31].

**Table 4:** Using hypnotics and the intensity of anxiety.

The Intensity of Anxiety (HADS—M) *	No hypnotic N (%)	Hypnotic N (%)	Total	*p*
0–7 points	176 (43.67%)	3 (16.67%)	179	*p* = 0.01
8–10 points	105 (26.05%)	3 (16.67%)	108	
>10 points	122 (30.27%)	12 (66.67%)	134	
Total	403	18	421	

* The intensity of anxiety disorders on the HADS-M scale: 0–7 points, no disorder; 8–10 points, borderline states; >10 points, disorders found [31].

## Data Availability

The data that support this study are available within the reference or available from the authors upon request.
